# Cell Models and Their Application for Studying Adipogenic Differentiation in Relation to Obesity: A Review

**DOI:** 10.3390/ijms17071040

**Published:** 2016-06-30

**Authors:** Francisco Javier Ruiz-Ojeda, Azahara Iris Rupérez, Carolina Gomez-Llorente, Angel Gil, Concepción María Aguilera

**Affiliations:** 1 Department of Biochemistry and Molecular Biology II, School of Pharmacy, Campus de Cartuja s/n, 18071, Institute of Nutrition and Food Technology “José Mataix”, Center of Biomedical Research, Avenida del Conocimiento s/n, 18016, University of Granada, Granada 18071, Spain; fruizojeda@ugr.es (F.J.R.-O.); azahararuperez@ugr.es (A.I.R.); gomezll@ugr.es (C.G.-L.); agil@ugr.es (A.G.); 2 Instituto de Investigación Biosanitaria ibs, Complejo Hospitalario Universitario de Granada/Universidad de Granada, Granada 18014, Spain; 3 CIBEROBN (Physiopathology of Obesity and Nutrition CB12/03/30038), Instituto de Salud Carlos III (ISCIII), Madrid 28029, Spain

**Keywords:** adipocytes, beige cells, brown adipose tissue, cell culture techniques, cell differentiation, in vitro techniques, obesity, white adipose tissue

## Abstract

Over the last several years, the increasing prevalence of obesity has favored an intense study of adipose tissue biology and the precise mechanisms involved in adipocyte differentiation and adipogenesis. Adipocyte commitment and differentiation are complex processes, which can be investigated thanks to the development of diverse in vitro cell models and molecular biology techniques that allow for a better understanding of adipogenesis and adipocyte dysfunction associated with obesity. The aim of the present work was to update the different animal and human cell culture models available for studying the in vitro adipogenic differentiation process related to obesity and its co-morbidities. The main characteristics, new protocols, and applications of the cell models used to study the adipogenesis in the last five years have been extensively revised. Moreover, we depict co-cultures and three-dimensional cultures, given their utility to understand the connections between adipocytes and their surrounding cells in adipose tissue.

## 1. Introduction

Obesity is one of the most important public health burdens both in developed and developing countries. It is characterized by an excessive accumulation of fat mass in white adipose tissue (WAT), which can occur through an increase in adipocyte volume (hypertrophy), number (hyperplasia), or a combination of both (hypertrophy–hyperplasia) [1].

Adipose tissue contains adipocytes in addition to a wide population of cells, such as macrophages, fibroblasts, pericytes, blood cells, endothelial cells, smooth muscle cells, mesenchymal stem cells (MSCs), and adipose precursor cells. All of these cells are located in the stromal vascular fraction (SVF), and the cell composition and phenotype of the SVF are usually different depending on the location of the adipose tissue and the adiposity [1,2].

Two types of adipose tissue, “white” and “brown”, have been described [1]. WAT is the principle site for safe energy storage, but it is also an endocrine organ that secretes cytokines and adipokines [3,4]. In the context of obesity, WAT is characterized by the presence of inflammation and oxidative stress associated with insulin resistance, which leads to systemic alterations such as metabolic syndrome [5,6]. In contrast, brown adipose tissue (BAT) is specialized in fat burning for heat generation and energy expenditure related to thermogenesis and to defend against cold and, eventually, obesity. Brown adipocytes promote energy expenditure via mitochondrial uncoupling protein 1 (UCP-1). An intermediate type of adipocytes that expresses UCP-1 has also been described. This type of adipocyte is referred to as beige or “*brite*” (brown in white) adipocytes and regarded as a non-classical/inducible brown adipocyte [7,8].

Adipocytes are derived from MSCs, which differentiate into lipoblasts, then into preadipocytes, and finally into mature adipocytes [9]. Adipocyte differentiation is a complex and multi-step process involving a cascade of transcription factors for key proteins that induce gene expression and lead to adipocyte development. During adipogenesis, fibroblast-like preadipocytes differentiate into lipid-laden and insulin-responsive adipocytes [10]. It is well known that peroxisome proliferator-activated receptor gamma (PPAR-γ), CCAAT/enhancer-binding proteins (C/EBPs), and sterol regulatory element binding protein (SREBP) transcription factors are the major determinants of adipocyte fate [11]. Interestingly, it has been reported that the isoform-2 of PPAR-γ (PPAR-γ2) is implicated in metabolic alterations such as obesity, insulin resistance, type 2 diabetes, and dyslipidemia. The PPAR-γ2 isoform is highly present in adipose tissue and functions to promote adipocyte differentiation and triacylglycerols storage [12].

Since the 1970s, the differentiation of fibroblast cell lines into adipocytes has been extensively studied [1]. The ability to study this process in a tissue culture dish has enabled the exploration of general cellular mechanisms. In the last few years, different cell culture models and protocols have become available to study adipocyte biology [13,14,15] as revised by Armani et al. [16] and Poulos et al. [17]. Mature adipocytes, MSCs, and preadipocytes can be easily isolated from adipose tissue homogenates and used for research purposes. Moreover, adipocytes may alsobe allowed to dedifferentiate into lipid-free multipotent cells, referred to as dedifferentiated fat (DFAT) cells.

The aim of the present work was to update the different animal and human cell culture models available for studying the in vitro adipogenic differentiation process as it is related to obesity and its co-morbidities. The main advantages, disadvantages, new protocols, and applications of the cell models used to study adipogenesis in the last five years have been extensively revised. Moreover, we depict co-cultures and three-dimensional cultures given their utility to understand the connections between adipocytes and their surrounding cells in adipose tissue.

## 2. Methodology

A comprehensive search of the relevant literature was performed in electronic databases: MEDLINE through PubMed (U.S. National Library of Medicine and the NIH). The following phrases were included in the search of the literature over the last five years (PubMed): differentiat* and adipocyte* and obesity and adipogen* and (“cell culture techniques” (MeSH) OR “cell line” (MeSH) OR preadipocyte* OR primary OR “adipocyte progenitor” OR “adipocyte precursor”). A total of 628 results in English were obtained, from which 568 articles were selected and categorized as works conducted with animal cell models and human cell models. Inclusion was based upon the use of cellular in vitro models to study the adipogenesis process related to obesity and its co-morbidities. Then, articles were classified according to the different cell types and applications. Additionally, previous original articles and reviews focusing on different cell lines useful for the study of adipogenesis in the context of obesity were carefully examined.

## 3. Animal Cell Models

Preadipose cells and mature adipocytes from different animals have been studied over the last several years. The most commonly used cells have traditionally been from rodents, although feline or porcine cells have also been used to a lesser extent. Although studies in animal models of obesity and related metabolic diseases offer significant insights, their applicability to humans is actually limited by the existing differences in their metabolism and physiology [18].

**Figure 1:**
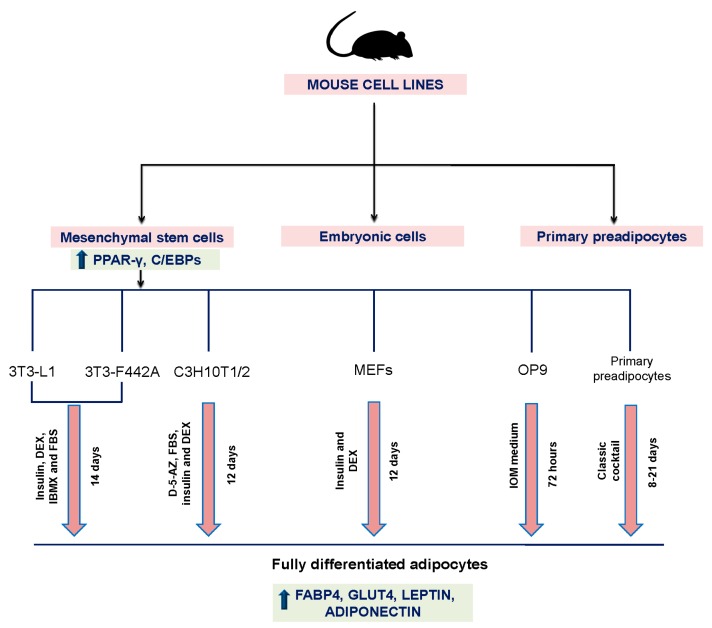
Mouse cell lines to study the adipogenesis process. DEX, dexamethasone; FBS, fetalbovineserum; IBMX, 3-isobutyl-1-methylxanthine; D-5-AZ, demethylating agent 5-azacytidine; IOMmedium, 10% FBS, 175 nM Insulin, 0.25 m MDEX, 0.5 m MIBMX, 2 mM l-glutamine, 100 U/mL penicillin, and 100 mg/mL streptomycin.

Primary preadipocytes are fibroblast-shaped cells that, under the appropriate conditions, can differentiate into mature adipocytes. Adipogenesis can be divided in two main phases: commitment and terminal differentiation. Adipogenic stimuli induce the differentiation in committed preadipocytes to an adipocyte phenotype prior to expressing some markers that include the PPARγ and C/EBP family of regulators. Once preadipocytes have committed to the adipogenesis program, a transcriptional cascade is activated that induces the expression of metabolic genes and adipokines associated with the adipocyte phenotypes, such as fatty acid-binding protein 4 (FABP4, also known as AP2), glucose transporter 4 (GLUT4, also known as SLC2A4), leptin, and adiponectin [19]. Murine preadipocytes have been commonly used to study various aspects of adipocyte biology and adipogenesis. Several major advantages of primary cultures are that they can be derived from various locations or depots and from animals of different ages to examine depot- or age-dependent adipogenic or secretory mechanisms, whereas preadipocyte cell lines are incapable of addressing these aspects [20]. Nevertheless, these models have several limitations such as they do not propagate in culture; they are more difficult to transfect with DNA; they have a huge triacylglycerol store that interferes with biochemical and microscopy analyses; they vary as a result of the genetics and conditions of the animals from which they are isolated; and the isolation procedure is tedious [21]. Additionally, differences exist between bovine, porcine, human, rat, and mouse preadipocytes such as 3T3-L1, 3T3-F442A, and C3H10T1/2 stromal cells (Table 1 and Figure 1).

### 3.1. 3T3-L1 Mouse Cell Line

The 3T3-L1 cell line is a well-established preadipose cell line that was developed from murine Swiss 3T3 cells [22]. The 3T3-L1 cells are derived from disaggregated 17- to 19-day-old Swiss 3T3 mouse embryos, which display a fibroblast-like morphology that, under appropriate conditions, can acquire an adipocyte-like phenotype [16,22,23]. Indeed, close to a third of the published articles in the last five years have described the use of 3T3-L1 cells for the study of adipogenesis and obesity-related characteristics.

Generally, to convert 3T3-L1 cells from their fibroblast phenotype into adipocytes, it is necessary to treat them with adipogenic agents, such as insulin, dexamethasone (DEX), and 3-isobutyl-1-methylxanthine (IBMX), which elevates the intracellular cAMP levels in the presence of fetal bovine serum (FBS) [29], at concentrations of 1 µg/mL, 0.25 µM, and 0.5 mM, respectively. Zebisch et al. reported that 3T3-L1 cells can differentiate within 10 to 12 days and persist for at least up to cell culture passage 10 using rosiglitazone (2 µM) as an additional adipogenic agent [25]. Moreover, Vishwanath et al. published a new method that promoted the differentiation of 3T3-L1 preadipocytes over a shorter span of time using a combination of DEX and troglitazone over fewer days compared to the combination of IBMX and DEX with the standard protocol. Moreover, by using DEX and troglitazone, the lipid droplet accumulation increased by 112%, and glucose transporter 4 (GLUT4) mediated a 137% higher glucose uptake compared to cells that were differentiated using the traditional method [24].

One of the main advantages of this cell line is that it is easier to culture and less costly to use than freshly isolated cells, such as mature adipocytes, even though freshly isolated cells allow for various comparisons, such as the in vitro evaluation of different in vivo conditions. Moreover, they can tolerate an increased number of passages and are homogeneous in terms of the cell population. Therefore, these cells provide a homogenous response following treatments and changes in experimental conditions [17].

Because of this, 3T3-L1 cells have been extensively over the last five years to evaluate the effects of compounds or nutrients on adipogenesis, to establish the underlying molecular mechanisms of adipogenesis and to evaluate the potential application of various compounds and nutrients in the treatment of obesity [30,31,32]. Particularly, compounds such as quercetin [33,34] and resveratrol [35,36] inhibit adipogenesis in 3T3-L1 adipocytes. Moreover, these cells have been used to describe the effect of melatonin [37], reactive oxygen species (ROS), or antioxidants [38] on adipogenic differentiation [39], and also, the role of some androgens such as testosterone that inhibit the adipogenic differentiation by activation of androgen receptor/β-catenin/T-cell factor 4 interaction in 3T3-L1 adipocytes [40]. Apart from that, 3T3-L1 cells have been useful to study the mechanisms of action during the differentiation process of several compounds or nutrients that have previously been shown in vivo to inhibit obesity [41]. Additionally, several endocrine disruptors and obesogenic compounds have also been evaluated during the differentiation of 3T3-L1 cells [42].

Furthermore, different gene silencing techniques such as siRNA and shRNA, together with different transfection procedures (adenovirus, lentivirus transfection, and plasmid electroporation) have been applied to study the function of different genes associated with adipogenesis in 3T3-L1 cells. In particular, inflammatory pathways, adipokine synthesis, and secretion of study enzyme’s function have been investigated through gene silencing in adipocytes [43,44,45,46,47]. These cells have also been useful for deciphering the biological role of several miRNAs, such as miRNA-195a, which plays an essential role in various cellular processes including proliferation and differentiation [48].

Finally, this cell line is useful in the study of co-cultures and three-dimensional cell cultures, as well as diverse studies of high-throughput screening of compounds [49,50].

However, the 3T3-L1 model has several limitations such as the time of initial subculture, in addition to the fact that adipogenic differentiation requires at least two weeks [51]. Moreover, when 3T3-L1 cells become confluent or they have been passaged extensively, they no longer differentiate into adipocytes; they are difficult to transfect; and because this cell line originated from a single clone, it fails to recapitulate the characteristics of primary cell culture models [21].

### 3.2. 3T3-F442A Mouse Cell Line

Another important cell line derived from murine Swiss 3T3 cells is the 3T3-F442A cell line, which displays a more advanced commitment in the adipocyte lineage than 3T3-L1 due to its isolation from a third selection of clones that converts into fat cells clusters of increased size and at a higher frequency. Thus, 3T3-F442A cells are capable of accumulating more fat than the 3T3-L1 cells, and an early exposure to glucocorticoids is not necessary to initiate the adipogenic differentiation of the 3T3-F442A cells [9,23]. Regarding in vitro adipogenic differentiation studies, this cell line has been significantly used less than the 3T3-L1 cells, despite the minimal differences between the differentiation protocols of both cell lines. Nevertheless, 3T3-F442A cells have also been used to study the effects of compounds during the differentiation process. Moreover, gene silencing through siRNA has been carried out to study the role of alkaline phosphatase in lipid metabolism and gene expression as well as the secretion of adipokines [52]. Additionally, this cell line has been used to study the effects of some drugs on adipocyte differentiation [53]. Finally, others have reported the effects of a variety of receptors and transcription factors during adipogenic differentiation [54].

In summary, 3T3-L1 and 3T3-F442A cell lines have been well established as good models for studying various aspects of adipogenesis in vitro since 1974, in spite of the described disadvantages of the 3T3-L1 cell line such as adipogenic differentiation time or the difficulty in transfecting these cells.

### 3.3. OP9 Mouse Cell Line

The OP9 mouse stromal cell line is a new adipocyte cell culture model that provides a tractable alternative for adipogenesis studies. This cell line was established from the calvaria of newborn mice genetically deficient in functional macrophage colony-stimulating factor. OP9 cells are mouse bone marrow-derived stromal cells that accumulate large triacylglycerol filled droplets after only seventy-two hours of adipogenic stimuli, making them a suitable model for high-throughput screening [21,55]. OP9 adipogenic differentiation is a PPAR-γ dependent process, and differentiated cells express PPAR-γ, CEBPα, CEBPβ, perilipin 1 (PLIN1), and PLIN4 proteins similar to other adipocyte models [55]. These cells have also been used in co-culture to support hematopoietic cell differentiation from embryonic stem cells [21,56]. Unlike 3T3-L1 cells, OP9 cells are able to differentiate into adipocytes after reaching confluence and are also able to be passaged for long periods of time in culture. Furthermore, they can be differentiated rapidly enough to detect protein expressed from transiently transfected DNA in fully differentiated adipocytes [21].

Three methods can be used to differentiate OP9 preadipocytes into adipocytes: the serum replacement method (SR), the insulin/oleate method (IOM), and the adipogenic cocktail method (AC). In the SR method, the cells are grown to confluence and then cultured for two additional days in OP9 propagation medium containing α-minimum essential medium eagle (α-MEM), FBS, l-glutamine, penicillin, and streptomycin. Then, cells are cultured up to four more days in serum replacement medium containing α-MEM, penicillin, and streptomycin. In the IOM method, when the adherent cells become confluent, the medium is replaced with insulin/oleate medium containing FBS, insulin, DEX, IBMX, l-glutamine, penicillin, and streptomycin. Finally, the AC method is very similar to that of the 3T3-L1 cells where the differentiation medium contains DMEM, FBS, l-glutamine, penicillin, streptomycin, insulin, DEX, and IBMX and the process lasts two days. Differentiated OP9 cells are maintained in OP9 propagation medium [57].

Regarding its applicability in recent studies, the OP9 cell line has been used to evaluate the effects of compounds or nutrients on the adipogenesis process. To this end, Seo et al. explored the mechanisms responsible for the anti-adipogenic activity of quercetin, and its’ effects on lipolysis in OP9 cells [58]. Similarly, Kim et al. investigated the inhibitory effects of *Pericarpium zanthoxyli* extract on the adipogenic differentiation of OP9 cells [59]. Another study showed that ascorbic acid, which has been demonstrated to be an adenylate cyclase inhibitor, inhibits adipogenesis in the OP9 cell line [60].

This cell line has also been used to study the role of oxidative stress on the adipogenesis process. The fullerene effects on adipogenesis-accompanying oxidative stress and inflammatory changes were also examined. Xiao et al. [61] demonstrated that hydrogen peroxide stimulates lipid accumulation in 3T3-L1 preadipocytes and that lipid uptake causes ROS generation in OP9 preadipocytes, both of which were then markedly suppressed with fullerene. Additionally, Saitoh et al. [62] investigated the effects of a novel polyhydroxylated fullerene derivate C_60_(OH)_44_, which is water-soluble with antioxidant properties, on intracellular lipid accumulation, intracellular ROS generation, lipid composition, and the protein expression of PPAR-γ in OP9 preadipocytes.

Conversely, Lane et al. investigated the feasibility of OP9 clonal derived cells as a model for rapid drug screening and the effect of gene knockdown on adipogenesis. They established a clonal population of OP9 cells, OP9-K, which differentiate rapidly, robustly, and reproducibly and compared the transcriptome of differentiating OP9-K cells with other models of adipogenesis. The transfection efficiency was 80% in OP9-K cells, and the cells differentiated rapidly and reproducibly into adipocytes. Moreover, they validated the OP9-K cells as an adipocyte model system for microarray analysis of the differentiating transcriptome [55]. One limitation of OP9 cells is that not every protocol may be optimized for adipocyte differentiation and manipulation, and also, that, when maintained at low cell density, OP9 cells adopt a spindly morphology and differentiate into adipocytes poorly.

In summary, the OP9 cell line has a clear potential use as a new model for the study of adipogenesis, and it could be useful for fast high-throughput studies.

### 3.4. C3H10T1/2 Mouse Cell Line

The C3H10T1/2 cell line was established in 1973 from 14- to 17-day-old C3H mouse embryonic stem cell precursors and has the capacity to differentiate into mesodermal cell types such as adipocytes, chondrocytes, osteoblasts, and myocytes. This cell line displays a fibroblast morphology similar to multipotent MSCs. Adipogenic differentiation can be induced by treatment with the demethylating agent 5′-azacytidine [9,26].

In the last five years, the main applications of C3H10T1/2 cells have focused on evaluating the effects of different compounds on adipogenesis and on investigating the molecular mechanisms related to adipogenic differentiation associated with obesity [63,64]. Specifically, as in the 3T3-L1 cell line, the role of miR-195a as regulator of adipocyte differentiation was studied in C3H10T1/2 cells [48]. Additionally, this cell line has been used for studying food contaminants such as tributyltin, which is an endocrine disrupting compound that promotes adipogenic differentiation in vitro [65]; some androgens, such as testosterone, inhibit adipogenesis in the C3H10T1/2 cell line through an androgen receptor-mediated pathway and β-catenin complex/T-cell factor-4 [40], and the androgen action activated a number of WNT target genes, including the Follistatin (*Fst*) gene (which binds and antagonizes native ligands of the TGF-β/Smad pathway) and cross communication with the Smad signaling pathway [66]; or in gene silencing studies by using shRNA [67]. Finally, the role that bone morphogenetic proteins (BMPs) play on adipogenesis was elucidated by Xue et al. [68] who performed an in vitro study using the C3H10T1/2 cells to investigate BMP4 and BMP7.

### 3.5. Primary Mouse Embryonic Fibroblasts (MEFs)

Primary mouse embryonic fibroblasts (MEFs) are derived from totipotent cells of early mouse mammalian embryos and are capable of differentiating into adipocytes with variable efficiency (usually 10%–70%), whereas most immortalized MEF lines do not differentiate unless a pro-adipogenic transcription factor such as PPAR-γ or C/EBPα is introduced [69]. MEFs present a number of properties that make them an attractive cell culture model. These cells are easy to establish and maintain, proliferate rapidly, and large numbers of cells can be produced from a single embryo within several days following explantation. Moreover, MEFs can be expanded through several passages. Similar to the primary cultures, MEFs have certain limitations as a consequence of their origin. Therefore, because of the cellular heterogeneity of embryonic tissue, the culture of these cells is often difficult, although steps can be taken to ensure a greater degree of homogeneity. Additionally, primary cultures of MEFs tend to reach senescence around passage 12 [28]. However, the adipogenesis of MEFs cells can be induced for eight days with a standard differentiation induction medium containing 0.5 mM IBMX, 1 µM DEX, 10 µg/mL insulin, 10 µM troglitazone, and 10% (*v*/*v*) FBS [27].

Recently, MEFs have been used to study adipogenesis in vitro as well as mechanisms related to obesity such as genes or transcription factor implicated in the adipogenesis process, signaling pathways in adipocytes, or the known fat mass and obesity-associated (*FTO*) gene. In this sense, MEFs derived from *FTO* overexpressing mice exhibited an increased potential for adipogenic differentiation, while MEFs derived from *FTO* knockout mice showed a reduced adipogenesis. Thus, fat pads from *FTO* mice fed a high-fat diet showed an increased number of adipocytes [70]. Conversely, Han et al. studied the role of the unfolded protein response (UPR), a protein associated with oxidative stress, in adipogenesis because UPR is expressed in adipose tissue [71]. Similarly, the role of deadenylase nocturnin (Noc), a protein found to regulate lipid metabolism and to control preadipocyte differentiation, in modulating early adipogenesis was studied in MEFs derived from 13.5-days-old embryos by Hee et al. [72]. Another study performed by Kim et al. [73] using MEFs to study the role of Makorin Ring Finger Protein 1 (MKRN1), which is a negative regulator of PPAR-γ2 in obesity, indicated that MKRN1 is a potential new therapeutic target in PPAR-γ related diseases. Recently, Braga et al. reported a novel role of *Fst* in regulation of energy/lipid metabolism and modulation of brown adipocytes and MEFs. In differentiated MEFs from *Fst*-KO mice, the induction of brown adipocyte proteins was attenuated, suggesting that Fst produced by adipocytes may act in a paracrine manner [74].

In summary, MEF cell lines appear to be a good model to study adipogenesis in vitro because it presents many advantages. However, as with all animal models, the main limitation is the origin (mouse embryos) and the particular physiological characteristics, which differ in a number of aspects from those of human adipocytes.

### 3.6. Porcine Primary Preadipocytes

Porcine preadipocytes are a much better model for the study of adipogenesis and obesity-related diseases compared to rodent cell models because of their higher similarity to human cells [75]. In porcine cell cultures, lipid-free preadipocytes are recruited during an early DEX period, and then, in the second period, insulin stimulates lipid accretion in the recruited preadipocytes. Although FBS was used for differentiation, it was eventually removed from differentiation medium of porcine cell cultures because it was shown to inhibit differentiation [19].

Porcine preadipocytes have been used as an adipocyte model to study the effects of different effectors on adipocyte dysfunction and metabolism. In this regard, Shu et al. described the role of phloretin, which promotes the differentiation of 3T3-L1 adipocytes, and demonstrated that phloretin enhances the lipid accumulation in a time-dependent manner in porcine primary adipocytes [76]. In another study, primary cultures of pig SVF cells were differentiated to study the effect of temperature on proliferation and differentiation [77]. Furthermore, retinol binding protein 4 (RBP-4) was observed to significantly suppress differentiation in porcine preadipocytes by decreasing the activation of insulin signaling pathways [78]. Pang et al. [79] studied the effects of Akt2 and sirtuin 1 (SIRT-1) on lipogenesis in porcine preadipocytes and its regulatory mechanisms.

This cell model has also been used to study the role of miRNAs. miR-125a, which promoted the differentiation of porcine preadipocytes upon inhibition, may provide new insights into pork quality improvement and obesity control [80]. In contrast, miR-199a, which is highly expressed in porcine subcutaneous fat deposits compared to several other tissue types, was observed to promote cell proliferation while attenuating the lipid deposition in porcine adipocytes [81]. Furthermore, miR-181a overexpression regulates adipogenesis by repressing the tumor necrosis factor-α in porcine preadipocytes, and it may become a new therapeutic target for anti-obesity drugs [82].

Conversely, porcine preadipocytes have been used to study different genes and mechanisms that inhibit adipogenesis. In this sense, Mai et al. described that BMP and activin membrane-bound inhibitor (BAMBI), inhibit adipogenesis through the wingless (Wnt)/β-catenin pathway in porcine preadipocytes [83]. Additionally, Pang et al. showed that forkhead box-1 (FOXO-1) and its regulation, through C/EBPβ and the phosphatidylinositol 3-kinase/glycogen synthase kinase 3-β (PI3K/GSK3β) signaling pathway, inhibited adipogenesis in porcine preadipocytes isolated from Bamei pigs (an obese breed) and large white pigs (a lean breed) [79].

### 3.7. Feline Primary Preadipocytes

Similar to porcine preadipocytes, feline preadipocytes have also been used to study adipogenesis in vitro. Adipogenesis can be induced in feline preadipocytes with preadipocyte medium containing insulin, DEX, biotin, pantothenate, IBMX, and a PPAR-γ agonist. After seven days, three-fifths of the medium is exchanged with adipocyte medium (lacking IBMX and the PPAR-γ agonist) [84]. Riedel et al. [84] investigated the presence of selected renin-angiotensin system (RAS) components in isolated feline adipocytes. Their results showed the existence of a potentially functional local RAS in feline adipose tissue that is differentially regulated during adipogenesis and dependent on the fat tissue depot and nutritional status. These findings could also be relevant for the understanding of the path-mechanisms in obese cats and dogs and could provide new approaches for the prevention and treatment of obesity-related diseases in cats.

In summary, animal cell models have been widely used to understand adipogenesis in vitro over the last several years because they are easier to isolate, less costly, and there are well-established differentiation protocols for the cell lines.

## 4. Human Cell Models

**Figure 2:**
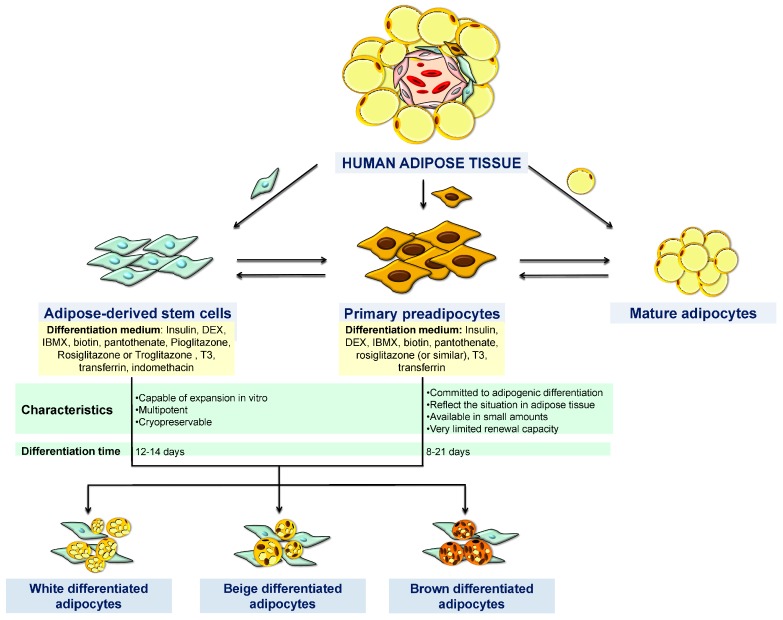
Human models to study the adipogenesis process. DEX, dexamethasone; IBMX, 3-isobutyl-1-methylxanthine; T3, triiodothyronine.

Although animal cell models have traditionally been the most frequently used for adipogenesis studies, human cells have been rapidly developed and are gaining importance in vitro studies. It is clear that results obtained with human cells are far more reliable than those from animal models because of their applicability towards human diseases such as obesity and its derived metabolic disturbances. In fact, human fat is the origin of these cell models, and it is derived from the SVF, which is formed by a mix of cells including preadipocytes, stem cells, endothelial cells, as well as immunological cells such as macrophages, neutrophils, and lymphocytes [85] (Figure 2).

### 4.1. Adipose-Derived Stem Cells (ASCs)

One of the most important cell types present in the SVF of adipose tissue is adipose-derived stem cells (ADSCs). Different names have been designated to describe this cell population isolated from adipose tissue: Adipose-Derived Stem/Stromal Cells (ASCs), Adipose Derived Adult Stem (ADAS) Cells, Adipose Derived Adult Stromal Cells (ADASC), Adipose Derived Stromal Cells (ADSC), Adipose Stromal Cells (ASC), Adipose Mesenchymal Stem Cells (AdMSC), Lipoblasts, Pericytes, Pre-Adipocytes, and Processed Lipoaspirate (PLA) Cells. Thus, the diverse nomenclature has led to significant confusion in the literature. To address this issue, the International Fat Applied Technology Society reached a consensus to adopt the term “Adipose-Derived Stem Cells” (ASCs) to identify the isolated, plastic adherent, multipotent cell population [86].

The presence of these perivascular cells in adipose tissue was discovered in the last century, when de novo fat formation was first observed [87]. Since then, strong research has been undertaken to characterize the nature of these multipotent stem cells, including their potential to differentiate into numerous cell types (adipocytes, chondrocytes, osteocytes, and myocytes) [86,88], as well as the best method for their isolation, culture, cryopreservation, and expansion [18,89,90]. Finally, ASCs have been distinguished from adherent bone marrow adult stem cells, which are known as MSCs and, recently, multipotent mesenchymal stromal cells (MMSCs) [89].

The main advantages of ASCs are their multipotency, their high expansion capacity, their ability to be passaged a number times, and their possibility of being cryopreserved for long periods of time [18]. Moreover, they reflect donor- and depot-specific characteristics, which is useful for assessing adipose tissue differences in proliferation or differentiation capacity.

Once differentiated into adipocytes, ASCs display phenotypic characteristics of genuine adipocytes; that is, freshly isolated ones. Specifically, they respond to physiologically relevant concentrations of hormones, including insulin and β-adrenergic agonists. Adipogenic differentiation of ASCs can be induced using culture medium supplemented with 0.5 mM IBMX, 50 μM indomethacin, and 0.5 μM DEX. The adipocyte medium needs to be changed every three days until mature adipocytes are obtained after 12–14 days of differentiation [89,91]. Interestingly, it has been proven critical to use a cAMP-elevating agent such as IBMX to obtain a correct adipogenic differentiation of ASCs [92], unlike in murine preadipocyte cell lines, where PPAR-γ agonists, DEX, and insulin are enough to promote adipogenesis.

In the last five years, ASCs have been used to study of the effect of different compounds on adipogenesis [93,94]. This cellular model has also been used for the characterization of molecules and cellular processes involved in adipogenesis [95,96,97,98]. Moreover, ASCs have served as a tool to investigate the role of different genes associated with adipocyte metabolism [99,100]. Finally, ASCs have also been used for the study of miRNAs [1,101] and browning, because they are capable of converting from white to brown adipocytes [102], which enables the study of the differential effect of certain molecules in white and brown adipogenesis such as p53 [103].

### 4.2. Primary Preadipocytes

Among the SVF cells, preadipocytes have proven to be an excellent model for the study of adipogenesis and fat cell biology. They can be easily obtained from adipose tissue and, under the appropriate conditions, differentiate into mature adipocytes.

ASCs and preadipocytes share many characteristics such as surface markers and have been used interchangeably in many studies where mixed preadipose cells obtained from the VSF are induced for adipogenesis. However, ASCs and preadipocytes also show some important differences. First, ASCs are Lin+/CD29+/Sca-1+/CD140a+, and committed preadipocytes are Lin−/CD29+/CD34+/Sca-1+/CD105−/CD117−/CD24+/CD140a−. Moreover, as Cawthorn et al. [86] explain in an excellent review, one of the main differences between both cell types is the expression of *PPAR-γ* by preadipocytes. Moreover, unlike ASCs, which retain a high proliferative and multiline-age-differentiation capacity, preadipocytes from the SVF are already committed to adipogenic differentiation, meaning that they can only differentiate into adipocytes.

Human primary preadipocytes are an excellent model for the study of adipocyte-related biology and obesity-related alterations because they reflect a situation close to that of adipose tissue. This is due to the presence of depot-specific properties, such as differences in the adipogenic capacity of visceral and subcutaneous preadipocytes, which may involve the existence of additional properties from the depot of origin. Indeed, the fact that they reflect characteristics from their donor makes them useful in studies assessing differences between individuals (obesity, weight-loss, age, etc.) [104,105,106,107,108]. Moreover, human preadipocytes do not require extensive proliferation in vitro to differentiate, suggesting that they may have already reached the necessary cell division state in vivo. Additionally, the fact that primary preadipocytes successfully differentiate in serum-free conditions allows for the study of specific compounds on adipogenesis, which could be inhibited by highly variable serum components [16].

Despite these advantages, human preadipocytes are usually available in small amounts and have a very limited renewal capacity. In an attempt to overcome this drawback, Darimont et al. [109] expanded the proliferative capacity of human SVF preadipocytes by adding telomerase activity through the co-expression of the h-TERT and E7 oncoprotein from the human papillomavirus type 16 (HPV-E7). It must be taken into account that a higher proliferative capacity is accompanied by a decline in the adipogenic potential as the number of passages increases. Thus, immortalized human preadipocytes require the addition of PPAR-γ agonists to properly accumulate lipids [110].

Preadipocyte adipogenic differentiation protocols are generally divided into an induction period and a maintenance period. The induction period usually lasts three to seven days and is characterized by the presence of insulin, IBMX, a PPAR-γ agonist or indomethacin, and DEX or cortisol (glucocorticoids that activate the glucocorticoid receptor GR). Interestingly, by prolonging the induction period from three to seven days, Lee et al. [111], found a significantly higher proportion of cells with adipocyte morphology together with higher adipogenic marker expression and improved metabolic phenotypes. Lee et al. [111] also studied the impact of 3% FBS on adipogenesis, which was inhibited in a dose-dependent manner, as were the responses to β-adrenergic-stimulated lipolysis. However, the rates of insulin-stimulated glucose uptake were higher in differentiated adipocytes with 3% FBS, whereas the sensitivity to insulin was almost unaltered.

Induction of gene expression in primary human preadipocytes has been achieved by different methods: lentiviral gene transfection [112], adenoviral delivery [113], or plasmid transfection [114]. Conversely, gene silencing has been generally achieved by siRNA delivery [115].

In the last five years, human preadipocytes have been used for many purposes. They have been proven useful for the study of precursor cell commitment and differentiation, a key process that has been found to be altered in hypertrophic obesity [105,113,114]. This cell culture model has also been widely used for the characterization of regulatory molecules implicated in the adipogenic differentiation process [116,117], including depot and disease-dependent differences in adipogenic capacity [104,106,108,118], as already mentioned above. Primary preadipocytes have also been employed to define differences and elucidate the origin and mechanisms behind alterations found in obesity [107,108,109,110,111,112,113,114,115,116,117,118,119,120]. The function of miRNAs on adipogenic differentiation and proliferation [101,121,122,123,124] has also been widely investigated in human preadipocytes.

In addition, the mechanisms by which endocrine disruptors increase adipogenesis and obesity has been explored with this cell model [125,126]. In contrast, the effects of different extracts and compounds inhibiting adipogenic differentiation have also been studied in primary human preadipocytes, suggesting potential treatments against obesity-related diseases [127,128].

Finally, primary human preadipocytes have also been used to validate [129] or refute findings from animal adipocyte models [130,131]. These studies imply that findings from studies in adipocyte cell models are extremely dependent on the exact model that is used. Thus, studies with the aim of elucidating human obesity-related disorders must always validate their findings using human cell models.

## 5. Brown/Beige Adipose Cell Lines

The interest in brown and beige adipocytes has been centered on the potential of these cell types to be used for the study of metabolic diseases such as obesity and its co-morbidities.

The major BAT depots in rodents are in the interscapular region embedded in and around deep back muscles. The interscapular BAT depots have also been found in human infants, which may decrease with age [132,133]. However, the presence of a metabolically highly active BAT in adult humans has been demonstrated [134]. Moreover, subsequent investigations have shown an inverse association between obesity and type 2 diabetes mellitus and the presence of active BAT [132,135]. Most brown fat cells originate from precursor cells in the embryonic mesoderm that also give rise to skeletal muscle cells and a subpopulation of white adipocytes. These precursors transiently express *Myf5* and *Pax7*, two genes that were previously thought to selectively mark skeletal myogenic cells in the mesoderm. The main activators described for the development of these cells are cold, thiazolidinedione’s, natriuretic peptides, thyroid hormone, fibroblast growth factor-21 (FGF-21), BMP7, BMP8b, and orexin. Beige adipocytes are located in the supraclavicular regions in humans and are interspersed within WAT subcutaneous fat in both mice and humans. Moreover, beige adipocytes do not have a history of *Myf5* expression, at least in the subcutaneous depot. It remains unknown whether beige adipocytes come from white adipocytes through trans-differentiation or if they arise from de novo differentiation and maturation of precursors [135]. The main activators described for these cells are the same as BAT activators, plus irisin and other myokines and cytokines, which display a selective action on beige adipocytes [135,136]. Within those myokines, a new small molecule, β-aminoisobutiryc acid, has been identified as a critical substance involved in the browning process of WAT [137].

Brown and beige adipogenic differentiation of a variety of animal and human cells can be achieved using specific medium containing supplements such as insulin, DEX, IBMX, rosiglitazone, and triiodothyronine (T3) in different concentrations [102,138,139,140,141,142] (Table 2).

Next, the main brown/beige preadipocytes and adipocytes from animals and humans developed over the last several years as adipocyte models to study the effects of different effectors in brown adipogenesis and some aspects related to obesity and browning in vitro are reviewed.

### 5.1. Primary Cell Models of Browning

Murine primary brown preadipocytes are primarily isolated from interscapular BAT from mice. These preadipose cells have been useful for studying the effects of compounds on BAT development by promoting brown adipogenesis [143]. In addition, the role of thermogenic genes or transcription factors capable of stimulating brown adipocyte differentiation has also been investigated [144,145].

As for human primary cells, they have also been useful for studying brown fat differentiation, with the primary advantage that human cells are used and, therefore, the results are more reliable. Additionally, there are other sources of primary adipocytes such as the progenitors isolated from human cervical fat, which were used by Lee et al. [146]. They reported that these cells were able to differentiate into adipocytes with either a brown adipocyte-like or white adipocyte phenotype and investigated the role of FGF-21. FGF-21, which is a cold-induced beiging adipokine capable of promoting a brown fat-like thermogenic program in WAT, could provide metabolic benefits of therapeutic relevance through browning of white adipose tissue.

### 5.2. Brown/Beige Differentiated Adipocytes

Brown adipocytes, like white adipocytes, are derived from multipotent stem cells. Similar to white adipogenic differentiation of different cell models such as 3T3-L1, 3T3-F442A, MSCs, human ASCs, SVF from WAT, and other cell lines, brown/beige adipogenic differentiation of these cell models has also been extensively reported over the last several years. Indeed, 3T3-L1 cells can differentiate into beige adipocytes with the appropriate differentiation cocktail that contains insulin, DEX, IBMX, rosiglitazone, and T3 [141,147,148,149]. In a similar way, the pluripotent C3H10T1/2 cells have served as a model for the study of the brown adipocyte developmental program by investigating the role of secreted frizzled-related protein 5 (SFRP5), which is a WNT protein inhibitor, in adipogenesis of white and brown adipocytes [150]. Moreover, C3H10T1/2 cells have been used to study the putative role of BMP4 in the differentiation of brown fat-like adipocytes [68]. Murine MSCs are also suitable candidates for BAT formation de novo. Sheyn et al., 2013 demonstrated that the transient overexpression of *PPAR-γ2* and *C/EBP-α* induces BAT because it mediates adipogenic differentiation [151].

Another murine cell line is the HIB-1B brown preadipocyte developed by Spiegelman (Harvard Medical School). The HIB-1B, which is a cell line derived from a brown fat tumor of a transgenic mouse, was the first established brown adipocyte cell line capable of expressing the brown fat-specific mitochondrial protein UCP-1 [152]. Kim et al. [153] studied the role of MKP3 as important factor in the regulation of brown adipocyte differentiation using H1B-1B cells.

As for human models, clones of brown and white preadipocytes from human neck fat have been characterized for their adipogenic and thermogenic differentiation capacity. Xue et al. [154] demonstrated the role of the positive UCP-1 regulators, PREX1 (phosphatidylinositol-3,4,5-triphosphate dependent Rac exchange factor 1) and EDNRB (endothelin receptor type B) for brown differentiation. Additionally, human ASCs are also able to differentiate into functional brown-like adipocytes upon the appropriate stimuli. ASCs from subcutaneous adipose tissue have been able to differentiate into “*brite*” adipocytes for the study of BMP4 on the browning process [138]. Similarly, MSCs from human bone marrow are also able to differentiate into white or brown adipocytes. Huang et al., 2011 investigated the role of PGC-1α (PPARG coactivator 1 alpha) in brown adipose differentiation. They reported that PGC-1α could mediate the differentiation of MSCs into brown adipocytes, which was accompanied by an increase in mitochondrial biogenesis and the up-regulation of UCP-1 expression. Because MSCs are multipotent stem cells, they demonstrated that PGC-1α inhibited the differentiation of MSCs into osteocytes under osteogenic conditions [155].

## 6. Cell Lines Representative of Various Diseases

### 6.1. Simpson-Golabi-Behemil Syndrome (SGBS) Cells

Simpson-Golabi-Behemil syndrome (SGBS) is an X-linked congenital overgrowth disease, characterized by macroglossia, macrosomia, renal and skeletal abnormalities, and an increased risk of embryonal cancer [156]. A preadipocyte cell strain was isolated from the stromal cells fraction of subcutaneous adipose tissue of an infant with SGBS. It is important to highlight that, technically, these cells are the only described, fully inducible preadipocyte cell line derived from humans. One of the main advantages of SGBS cells is that they can be considered as an unbounded source of homogenous and fully inducible cells [157,158]. Induction of differentiation from SGBS cells to adipocytes is performed in the presence of insulin, T3, cortisol, and the PPAR-γ agonist BRL49653 [157]. Moreover, adipogenic differentiation is carried out in a serum- and albumin-free medium [159].

SGBS cells have the capacity for adipogenic differentiation and, once differentiated, show a gene expression profile comparable to that of mature human fat cells [157]. Fully differentiated SGBS cells present a more similar morphology, transcript level, and biochemical function to primary omental adipocytes than 3T3-L1 cells. Moreover, co-culture of the esophageal adenocarcinoma cell line (OE33) cells with SGBS or primary omental adipocytes induced the differential expression of genes involved in adhesion, angiogenesis, and invasion and metastasis [160]. In addition, using SGBS cells as preadipocyte and adipocyte models has allowed for the development of a novel high-content analysis procedure for direct visualization and quantification of adipogenesis and adipoapoptosis by laser scanning cytometry [161].

Recently, the link between the metabolic and gene regulatory networks has been studied using the SGBS cell line and endothelial cells using experimental and computational analyses. Their results reveal the convergence of miRNAs and transcription factors within the branched amino acid metabolic pathway, which provides a possible explanation for its down-regulation in obese and diabetic conditions [162].

In conclusion, the SGBS cell line can be considered as an appropriate model for the study of various aspects of adipocyte differentiation in humans. However, we need to bear in mind that they are derived from a specific syndrome that appears to arise as a result of either deletions or point mutations within the *glypican 3* (*GPC3*) genes or by mutations in another currently unknown genes [159].

### 6.2. LiSa-2 Cells

Another cell model that might serve as a tool to investigate the effects of chemical or food compounds on adipogenesis is the LiSa-2 cell line, a stable cell line derived from a poorly differentiated, pleomorphic liposarcoma. This cell line retains the potential to undergo adipose differentiation [163]. Differentiated LiSa-2 cells display multiple small lipid droplets [164]. Only a few studies have used this cell line. Two studies were focused on the effect of HIV-protease inhibitors and catalase function [165,166]. In addition, another study using these cells described that knockdown of the COP9 signalosome (CNS) elevates the C/EBP homologous protein, which retards adipogenesis [88].

Differences in gene expression between isolated adipocytes and LiSa-2 cells have been observed, particularly in genes involved in fatty acid metabolism. These differences may be explained by the inability of LiSa-2 cells to develop into fully unilocular adipocytes, to their immortality, which may be the cause of the continuous cell growth that is observed during their differentiationand to the lack of essential factors such as nutrients or signaling molecules that are present in vivo and missing in vitro [164].

## 7. Co-Cultures and Three-Dimensional Cultures (3D)

Co-cultures and three-dimensional (3D) cultures of adipocytes with other cell types (i.e., endothelial cells, macrophages, and muscle cells) are crucial tools for understanding the multiple metabolic connections between fat and other tissues. These studies provide a more real insight into the factors and pathways that may be the target of new pharmacological interventions against obesity and its co-morbidities [16].

Most co-culture studies using adipocytes have been designed to investigate the relationship between obesity and insulin resistance or inflammation. This can be evaluated by co-culturing macrophages and adipocytes because the direct cell-cell contact is capable of inducing an inflammatory response in adipocytes. Accordingly, a co-culture of 3T3-L1 adipocytes and macrophages developed by Huang et al. [49] suggested that phloretin (PT) and phlorizin (PZ), which are natural drugs used to treat diabetes and inhibit adipocyte differentiation, increased lipolysis in adipocytes, and also, suppressed the macrophage inflammatory response that is stimulated by conditioned medium from 3T3-L1 cells. Similarly, Kim et al. [165] carried out astudy of the role of esculetin, an anti-inflammatory compound isolated from natural plants, in a co-culture with 3T3-L1 adipocytes and macrophages. This study demonstrated that esculetin exhibited anti-inflammatory properties by inhibiting the production of some cytokines such as TNF-α and MCP-1 in the interaction between adipocytes and macrophages through hemooxigenase-1.

Additionally, an established human model system that combines the THP-1 monocytic cell line, which originates from an acute monocytic leukemia, and the preadipocyte cell strain SGBS was proposed as a useful model to study adipose inflammation in vitro. This co-culture model represents an inexpensive, highly reproducible human adipose tissue inflammation system, which can be extended for use with primary human macrophages and fat cells [166].

Trying to better resemble the adipose tissue cell plasticity, Chazenbalk et al., 2011 carried out an in vitro co-culture with ASCs and adipose tissue macrophages (ATMs) resulting in a robust proliferation of preadipocytes. Moreover, these new preadipocytes were observed to rapidly turn into adipocytes. They demonstrated that the co-culture of adipocytes with ATMs and ASCs increased the formation of new preadipocytes, increased lipid accumulation, and C/EBPα and PPAR-γ gene expression [167]. Regarding the adipogenesis process, it has been reported a co-culture method of preadipocytes with primary subcutaneous and visceral adipocytes to identify molecules that regulate adipocyte differentiation, such asSlc27a1 (Solute Carrier Family 27 (Fatty Acid Transporter), Member 1), vimentin, Ceruloplamsine and Ecm1 (extracellular matrix protein 1) promoted adipocyte differentiation, whereas Got2 (a known cell-surface fatty acid transporter) or Interleukin-1 receptor like 1 decreased the differentiation. These findings demonstrated a regulation of adipocyte differentiation through positive or negative and autocrine feedback loop mechanisms [168].

The biological significance of 3D cell culture in understanding cellular behavior and function is highlighted by studies with various cell types. Consequently, 3D adipocyte cultures have been developed to better understand the role of adipocytes in adipogenesis and the treatment of obesity over the last several years. Regarding inflammation, a 3D spheroid organization of adipose cells was reproduced by culturing 3T3-L1 preadipocytes on an elastin-like polyethyleneimine (ELP-PEI)-coated surface. This work investigated the cellular responses to a pro-inflammatory stimulus, indicating a more differentiated phenotype in 3D spheroid cultures relative to two-dimensional (2D) monolayer analogs. Therefore, this 3D spheroid model with enhanced adipogenic differentiation features a platform for elucidating the key phenotypic responses that occur in pro-inflammatory microenvironments that characterize obesogenic states [169].

Recently, significant efforts have been made to understand the biology of BAT and its potential as a new therapeutic target for obesity. In this sense, Unser et al. [170] created “brown-fat-in-microstands” by encapsulating brown preadipocytes and pluripotent stem cells in 3D alginate hydrogel microstrands and directly differentiating them into functional brown adipocytes. Accordingly, they used mouse embryonic stem cells, which were used as the model to test the feasibility of 3D brown adipogenesis in alginate microstrands. The microstrands expressed the brown adipocyte-defining marker UCP-1 and exhibited characteristics of brown adipocyte activation in response to β-adrenergic agonists.

Finally, some efforts have been made to provide the cells with a more physiologically relevant environment by using surface-structure and 3D culture systems. Thus, Brännmark et al. [171] investigated the effect of using aligned and randomly oriented polycarprolactone fibers matrices for large cell populations of ASCs differentiated into adipocytes by measuring proliferation, glucose uptake, gene expression, and lipolysis. Moreover, those results were compared with human primary mature adipocytes demonstrating an increased maturity of this adipocyte cell model using ASCs differentiation on aligned polycaprolactone fiber matrices compared to classic cultured cells.

## 8. Conclusions

The present review focuses on the available cellular models useful for evaluating the adipogenesis process and adipocyte differentiation in vitro related to obesity and adipocyte dysfunction. Recently, new cell lines and protocols have appeared to improve adipocyte culture, such as the OP9 cell line and other models to study the brown/beige adipocytes. However, the 3T3-L1 cells remain to be the most commonly used cell model for studying adipogenesis in vitro because the protocols for these cells are highly developed and standardized. In contrast, results arising from these studies are not as useful for applications on human health as human cell lines, given the physiological and metabolic differences between species. In particular, human preadipocytes and ASCs have become excellent models for studying adipogenesis and obesity-related metabolic alterations as well as for studying adipocyte renewal and expansion and donor and depot-specific differences. Finally, co-cultures and 3D cultures are essential for the better understanding of the connections between adipocytes and their surrounding cells in both health and disease situations. The main limitation of the current models is the great diversity of protocols for some cell lines, such as, for instance, the precise concentration of the compounds needed in the adipogenic differentiation cocktail.

## Figures and Tables

**Table 1:** Mouse cell lines to study the adipogenesis process.

Cell Model	Source	Differentiation Time	Characteristics	Differentiation Cocktail	Articles in the Last 5 Years
3T3-L1	Murine Swiss 3T3 cells from embryos	14 days	Easier and less costly to use than freshly isolated cells Stand a high number of passages Homogenous response to treatments and experiments	Insulin, DEX, IBMX and FBS (Green and Meuth 1974 [ 22]) DEX and troglitazone (Vishwanath et al. [24]) Rosiglitazone (Zebisch et al. [25])	392
3T3-F442A	Murine Swiss 3T3 cells	14 days	More advanced commitment towards adipocyte differentiation than 3T3-L1 cells	Insulin, DEX, IBMX and FBS (Green and Kehinde [23])	7
C3H10T1/2	Mouse embryonic stem cell precursor	12 days	Homogeneous population of multipotent cells More commitment of stem cells towards the adipocyte lineage	Demethylating agent 5-azacytidine and 10% FBS, insulin and DEX (Reznikoff et al. [26])	13
OP9	Mouse stromal cell	72 h	Fast adipogenic differentiation (72 h) Confluent after many passages Long periods in culture suitable for high-throughput screening	IOM medium (Wolins et al. [21])	6
MEFs	Mouse embryonic fibroblasts	14–15 days	Unlimited, undifferentiated proliferation in vitro	Fei medium (Fei et al. [27]) Petrov medium and Bauters medium [28]	9

Abbreviations: Bauters medium, 1.7 μM insulin, 1 μM DEX, 0.5 mM IBMX, and 5 μM rosiglitazone; DEX, dexamethasone; FBS, fetal bovine serum; Fei medium, 0.5 mM IBMX, 1 µM DEX, 10 µg/mL insulin, 10 µM troglitazone, and 10% FBS; IBMX, 3-isobutyl-1-methylxanthine; IOM medium, 10% FBS, 175 nM insulin, 0.25 mM DEX, 0.5 mM IBMX, 2 mM l-glutamine, 100 U/mL penicillin, and 100 mg/mL streptomycin; Petrov medium, 1 µmol/L DEX, 0.5 mmol/L IBMX, 5 µg/mL insulin, and 0.5 µmol/L rosiglitazone.

**Table 2:** Brown/beige adipogenic differentiation cocktails.

	Elsen et al [138]	Than et al. [139]	Li et al. [140]	Pisani et al. [102]	Hiroki et al. [141]	Gburcik et al. [142]
Cells	hASC (Subcutaneous AT)	Rat Primary Preadipocytes	Mice Primary Cultures of White and Brown Adipocytes	hMADS	3T3-L1	Brown Adipocytes (3129/Sv Strain of Mice)
*Adipogenic induction*						
Insulin	66 nM	860 nM	850 nM	850 nM	1.72 nM	2.4 nM
Dexamethasone	5.1 nM	1 nM	1 mM	1 μM	0.25 μM	
Indomethacin	125 μM		125 nM			
IBMX	0.5 mM	0.5 mM	0.5 mM	0.5 mM	0.5 mM	
Rosiglitazone	0.5 μM	1 mM	1 μM	0.1 μM	1 μM	1 μM
FBS	10%	10%				10%
T3	1 nM	1 nM	1 nM	0.2 nM	50 nM	
Cortisol	100 nM					
Transferrin				10 μg/mL		
Apo-transferrin	10 mg/mL					
Gentamycin	50 mg/mL					
Troglitazone	5 μM					
Ascorbate						25 g/mL
l-Glutamine						4 mM
Differentiation Days	14	8	7	14–16	8	6

FBS, fetal bovine serum; hASC, human adipose-derived stem cells; hMADS, human mesenchymal adipose-derived stem cells; IBMX, 3-isobutyl-1-methylxanthine; T3, triiodothyronine.
